# Intestinal barrier permeability: the influence of gut microbiota, nutrition, and exercise

**DOI:** 10.3389/fphys.2024.1380713

**Published:** 2024-07-08

**Authors:** Tetiana R. Dmytriv, Kenneth B. Storey, Volodymyr I. Lushchak

**Affiliations:** ^1^ Department of Biochemistry and Biotechnology, Vasyl Stefanyk Precarpathian National University, Ivano-Frankivsk, Ukraine; ^2^ Research and Development University, Ivano-Frankivsk, Ukraine

**Keywords:** tight junction proteins, ROS, TLR4, inflammation, NF-κB, HIF-1α

## Abstract

The intestinal wall is a selectively permeable barrier between the content of the intestinal lumen and the internal environment of the body. Disturbances of intestinal wall permeability can potentially lead to unwanted activation of the enteric immune system due to excessive contact with gut microbiota and its components, and the development of endotoxemia, when the level of bacterial lipopolysaccharides increases in the blood, causing chronic low-intensity inflammation. In this review, the following aspects are covered: the structure of the intestinal wall barrier; the influence of the gut microbiota on the permeability of the intestinal wall via the regulation of functioning of tight junction proteins, synthesis/degradation of mucus and antioxidant effects; the molecular mechanisms of activation of the pro-inflammatory response caused by bacterial invasion through the TLR4-induced TIRAP/MyD88 and TRAM/TRIF signaling cascades; the influence of nutrition on intestinal permeability, and the influence of exercise with an emphasis on exercise-induced heat stress and hypoxia. Overall, this review provides some insight into how to prevent excessive intestinal barrier permeability and the associated inflammatory processes involved in many if not most pathologies. Some diets and physical exercise are supposed to be non-pharmacological approaches to maintain the integrity of intestinal barrier function and provide its efficient operation. However, at an early age, the increased intestinal permeability has a hormetic effect and contributes to the development of the immune system.

## 1 Introduction

The intestinal wall is a complex system consisting of four layers: mucosa, submucosa, muscularis, and serosa. The term “intestinal barrier” emphasizes the protective component of the intestinal wall, whereas intestinal permeability is a measurable characteristic of the functional status of the intestinal barrier (Bischoff et al., 2014). The wall provides selective absorption of nutrients and other components of the intestinal lumen. At the same time, the intestinal barrier protects the body from the entrance of unwanted foreign substances, food particles, microorganisms, and their components. In normally functioning organisms, the permeability of the intestinal wall is tightly controlled but its disturbance, if not adequately fixed, can lead to many, if not most, acquired pathologies (Gieryńska et al., 2022).

The gastrointestinal tract (GIT) is inhabited by diverse microbes called gut microbiota forming very dynamic community. The “Old Friends Hypothesis” suggests that people co-evolved with many microbes that, in addition to many physiological functions, also stimulate the development of the immune system and regulate its operation (Rook, 2023). Microbial antigens are under constant surveillance by the enteric immune system. Regulatory immune T cells are responsible for maintaining immune tolerance of homeostatic gut microbiota (Wu and Wu, 2012). However, increased intestinal permeability can promote translocation of luminal bacteria and microbial-associated molecular patterns, in particular, lipopolysaccharides (LPS) from the gut into bloodstream, triggering the development of endotoxemia and chronic low-intensity inflammation (Vanuytsel et al., 2021). Diet-induced endotoxemia is defined as metabolic endotoxemia. For example, Cani et al. (2007) established that a high-fat diet chronically increased plasma LPS concentrations two-to threefold.

Endogenous LPS are constantly released as a result of the death of Gram-negative bacteria in the gut. At increased intestinal barrier permeability, LPS are absorbed into the portal bloodstream, from where they are transported by lipoproteins directly into the liver, forming the gut-liver axis. Further, they are metabolized by liver enzymes and excreted with bile. However, if their degradation or biliary excretion are impaired, LPS can reach the systemic circulation, where they bind to Toll-like receptor 4 (TLR4) on leukocytes, endothelial cells, and platelets, causing arterial inflammation. Ultimately, this leads to activation of blood coagulation and thrombus formation, which demonstrates that LPS-induced inflammation associated with increased intestinal wall permeability may be involved in the development of atherosclerosis and thrombotic diseases (Violi et al., 2023). In general, disruption of intestinal barrier function is involved in many GIT-related and unrelated diseases, including inflammatory bowel disease, metabolic dysfunction-associated liver disease, bile acid malabsorption, celiac disease, type I diabetes, obesity, schizophrenia, and others (Vanuytsel et al., 2021). Potentially, this could be overcome by a non-pharmacological intervention based on diet and exercises (Pražnikar et al., 2020; Ordille and Phadtare, 2023) which promote a healthy gut ecosystem and alleviate the symptoms of many pathologies.

In this review, we describe the structure of the intestinal wall and molecular mechanisms of the pro-inflammatory response caused by bacterial invasion due to the disturbance of the intestinal wall permeability, as well as influences of the gut microbiota, diet, and exercises on the permeability of the intestinal wall. Specific diets and regular low- and moderate-intensity exercises are proposed as effective non-pharmacological approaches to maintain integrity of intestinal wall and its efficient operation. However, at an early age, controlled leakage of the intestine may be necessary to trigger the development of immune system via hormetic mechanisms.

## 2 The structure of the intestinal barrier

**FIGURE 1:**
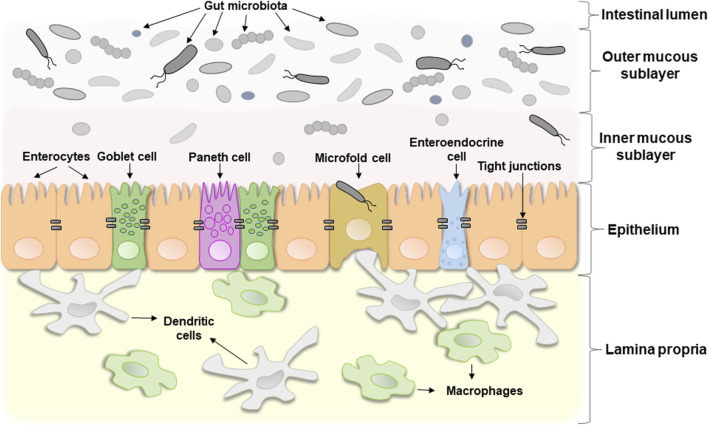
The schematic structure of the intestinal barrier. For details see the text.

The term “intestinal barrier” emphasizes the barrier function of the intestinal wall which protects organism against invading by bacteria or other microorganisms and potentially toxic components of microorganisms. In fact, it is a complex selective physical barrier that separates the internal environment of the body from the contents of the intestinal lumen (Bischoff et al., 2014). Figure 1 shows a schematic structure of the intestinal barrier. It consists of several layers: i) a mucous layer including inner and outer mucous sublayers inhabited by commensal microorganisms in a different extent, ii) a single layer of epithelial cells, and iii) the lamina propria, which consists of immune cells that instantly react to the invasion of foreign substances (Schoultz and Keita, 2020).

The mucous layer, that consists mainly of a mesh polymer called mucin, is located on the side of the intestinal lumen. It is associated with community of commensal microorganisms, including bacteria, fungi, viruses, and parasites, that form the individual microbial community (Chelakkot et al., 2018). A change in the microbial composition that causes a sharp imbalance between beneficial and potentially pathogenic bacteria, including changes in its functional composition, metabolic activity or changes in their local distribution, is called dysbiosis or dysbacteriosis. The latter usually results from loss of beneficial bacteria, overgrowth of potentially pathogenic bacteria, or loss of overall bacterial diversity. This disrupts the homeostatic balance of the intestinal microbiota and has a negative impact on the host’s health. In particular, dysbacteriosis is implicated in a wide range of diseases (DeGruttola et al., 2016). Indeed, Walter et al. (2020) inform that 95% of published studies on germ-free rodents transplanted with human fecal microbiota reported transmission of pathological phenotypes to recipient animals and many studies reported causal relationships. However, the role of gut microbiota in the development of various diseases is often greatly overestimated and needs a more critical analysis.

Bacteria of the phyla Bacteroidota (older name Bacteroidetes) and Bacillota (older name Firmicutes) usually dominate in typical healthy adults. Bacillota account for up 65% of the composition of the gut microbiota, with Bacteroidota—16%, Actinomycetota (older name Actinobacteria)—9%, and Pseudomonadota (older name Proteobacteria)—5% (Belizário and Faintuch, 2018). The density of gut microbiota distribution varies along the GIT, which can be divided into the oral cavity, esophagus, stomach, small intestine, and colon. Along this longitudinal axis from oral cavity to the colon, the microbial load and its diversity increase. This microbial gradient depends on many factors, including pH, concentrations of oxygen and antimicrobial peptides, level of bile acids, mucus thickness, and transit time. For example, the lowest level of oxygen, amounts of antimicrobial peptides and bile acids, as well as sufficient mucus thickness, moderate pH, and the longest transit time in the colon create the most optimal conditions for coexistence with commensal microbes. While the shortest transit time in the oral cavity and esophagus, the highest concentration of antimicrobial peptides and bile acids in the small intestine, and the lowest pH in the stomach limit bacterial colonization, contributing to the formation of a microbial load gradient along the GIT (Scheithauer et al., 2016; Kennedy and Chang, 2020; Mailhe et al., 2018). In addition, each part of the GIT is characterized by a special biodiversity. For example, the phylum Pseudomonadota, as well as such families as Streptococcaceae and Veillonellaceae (phylum Bacillota) are more common in the small intestine. Whereas the distal part of the small intestine and the colon are characterized by the dominance of the families Bacteroidaceae (phylum Bacteroidota), Lachnospiraceae, and Ruminococcaceae (phylum Bacillota) (Jensen et al., 2023). However, the biodistribution of intestinal microbiota changes not only along the longitudinal axis, as we mentioned above, but also transversely. For example, the families Bacteroidaceae, Enterococcaceae, and Lactobacillaceae are more common in the intestinal lumen, while the families Lachnospiraceae, Ruminococcaceae are more common in the mucosa (Daniel et al., 2021).

In general, commensal microorganisms participate in host digestion, biosynthesis of vitamins, production of short-chain fatty acids (SCFAs) and bacteriocins, development of the host’s immune system, and functioning of the gut-brain axis. The interaction of gut microorganisms and their waste products with the intestinal immune system at an early age helps to distinguish self from non-self (invaders). In this way, gut microbiota trains immune system and takes a direct part in its development (Belizário and Faintuch, 2018). In addition, gut microorganisms are part of so-called gut-brain axis, a bidirectional communication between the gut and the brain (Carabotti et al., 2015). Intestinal microorganisms affect the host’s nervous system and vice versa. GIT is densely innervated by a network of 200–600 million neurons that form the enteric nervous system, which interacts with gut microbes and the gut immune system (Geng et al., 2022). Interesting, transplantation of fecal microbiota from patients with depression into a microbiota-deficient rat model caused behavioral and physiological features that are characteristic of depression (Kelly et al., 2016). This research demonstrated the importance of the microbial community in the operation of the gut-brain axis and the development of disorders of the nervous system.

The second layer, the intestinal epithelium, consists of a single layer of several specialized epithelial cells, such as enterocytes, Goblet cells, Paneth cells, enteroendocrine cells, and microfold cells (Figure 1). Enterocytes form the basis of the intestinal epithelium and play a main role in the absorption of all consumed nutrients. Goblet cells constitute about 10% of specialized epithelial cells. They secrete mucus to protect the intestinal wall from digestive enzymes (Kim and Ho, 2010). Paneth cells contain secretory granules filled with antimicrobial peptides, that are secreted in low amounts constitutively and provide the antimicrobial properties of the intestinal mucosa. Under certain conditions, their secretion can increase dramatically (Yokoi et al., 2019). Enteroendocrine cells produce hormones regulating secretion of digestive enzymes and insulin, peristalsis of the intestine, satiety, and immune response (Bonis et al., 2021). Microfold cells transport bacteria and antigens from the epithelium to enteric immune cells that either activate or suppress the immune response (Jung et al., 2010). All these cell types collectively contribute significantly to gut homeostasis.

The third layer, lamina propria, is located under the epithelium and forms the enteric immune system that consists of a large number of leukocytes with macrophages and dendritic cells being the dominant cell types (Shemtov et al., 2023). Resident intestinal macrophages are located in close proximity to the gut microbiota, with which they often interact. They play a key role in immune sampling of luminal bacteria, contributing to the maintenance of intestinal homeostasis and regulated immune response. The meeting of intestinal macrophages with commensal microorganisms under homeostatic conditions does not trigger the development of a clear inflammatory reaction, that is associated with constitutive IL-10 signaling. However, under certain conditions, monocytes accumulate in the intestines and differentiate into highly sensitive pro-inflammatory macrophages. The latter in response to stimulation by intestinal microbial antigens, particularly LPS, upregulate the production of proinflammatory cytokines (e.g., IL-1β, IL-6, TNF) and reactive oxygen species (ROS), contributing to the enhancement of pro-inflammatory reactions (Hine and Loke, 2020). In addition, under inflammatory conditions both, immune and epithelial cells, can release proteases into lamina propria, which may degrade the layer of epithelial cells lining the gut (Van Spaendonk et al., 2017). In turn, this disrupts the gut barrier function and may result in acute and chronic inflammatory states (Hine and Loke, 2020).

In addition, it is worth mentioning the so-called gut-vascular barrier (GVB), which is formed by endothelial cells surrounded by enteric glial cells and pericytes. Spadoni et al. (2015) established that fluorescein isothiocyanate (FITC)-dextran of 4 kD freely diffused through the GVB, whereas FITC-dextran of 70 kD could not, whereas infection with *Salmonella enterica* disrupted GVB and promoted 70 kD FITC-dextran crossing GVB. Thus, the GVB barrier controls the translocation of antigens of various sizes from the gut into the bloodstream and, in particular, prevents the penetration of intestinal microbiota into the portal bloodstream (Spadoni et al., 2015).

## 3 Intestinal permeability

Semi-permeability or selective permeability is a crucial feature of the intestinal wall. It limits penetration of pathogens but allows the permeability of nutrients, water, and ions. Endogenous (e.g., inflammation) and exogenous (e.g., diet components, toxicants, or drugs) factors can increase intestinal permeability and cause the formation of a so-called “leaky gut.” The latter is characterized by the penetration of food antigens, commensals, or pathogenic bacteria into the blood, causing the development of inflammation (Vanuytsel et al., 2021). Some diseases can also act as a disruptor factor of the intestinal barrier. For example, several studies show that hyperglycemia, a key feature of diabetes, induces intestinal barrier dysfunction (Thaiss et al., 2018; Dubois et al., 2023). Prolonged exposure to glucose at high levels increases migration capacity of human colonic cell line Caco-2, resulting in layers appearing less organized than under physiological conditions. In particular, this is associated with decreased expression of tight junction (TJ) proteins, which contributes to the disruption of the structural network associated with them and an increase in the permeability of the intestinal barrier (Dubois et al., 2023). In turn, this contributes to the penetration of luminal bacteria, and the development of dysbacteriosis resulting in inflammation. For example, Harbison et al. (2019) showed that children with type I diabetes have gut microbiota dysbiosis associated with increased intestinal permeability. In particular, lower microbial diversity, lower numbers of anti-inflammatory bacterial species, and SCFA-producing bacteria were observed, and these changes were not explained by differences in diet. Thus, some diseases, including diabetes, can also play the role of disruptors of the intestinal barrier.

Mucus and epithelium are the most important components of the intestinal barrier that limit the development of inflammation. The mucous layer consists of two sublayers (Figure 1). The outer sublayer is thick and loose. It is inhabited by a large number of commensal microorganisms that form colonies, and under healthy conditions pathogenic bacteria cannot outgrow them or penetrate further. In other words, homeostatic microorganisms efficiently compete with potentially pathogenic ones and prevent their excessive proliferation. The inner sublayer, on the contrary, is solid and contains only a few microbes (Usuda et al., 2021). The gut microbiota plays a major role in changing the composition of mucus, regulating its synthesis and degradation.

Epithelial cells are connected by TJ proteins (Lee et al., 2018) which regulate the absorption of water, ions, and dissolved substances. They include two functional categories of proteins: integral transmembrane proteins, located at the border of adjacent cell membranes, and adaptive peripheral membrane proteins that connect integral proteins with the actin cytoskeleton. The former includes occludin, claudins, junctional adhesion molecules, and tricellulin whereas the latter include zonula occludens-1 (ZO-1), ZO-2, and ZO-3 (Lee et al., 2018). The gut microbiota can influence the expression and localization of all of these TJ proteins.

### 3.1 Influence of the gut microbiota on tight junction proteins

TJ proteins regulate the rate of paracellular transport including the transport of consumed nutrients via the path between neighboring epithelial cells. In electron micrographs TJ proteins look like points of fusion of the membranes of neighboring cells where there is no intercellular space in these places (Gonzalez-Mariscal et al., 2003). They play the role of sensors of environmental conditions that dynamically regulate the paracellular transport of solutes (Ulluwishewa et al., 2011). Dysregulation of TJ proteins can lead to excessive permeability of the intestinal barrier.

Bacteria can change the expression and distribution of TJ proteins and thus affect intestinal permeability. For example, some pathogenic strains of *Escherichia coli*, including *E. coli* O157:H7 strain which causes bloody diarrhea, produce toxins such as Shiga toxins (STx). The latter suppress protein biosynthesis and contribute to the development of hemolytic uremic syndrome, which is a life-threatening complication. Pradhan et al. (2020) found that STx2a decreases the expression of TJ proteins such as ZO-2, occludin, and claudin-1 (Pradhan et al., 2020). However, this strain requires the presence of non-pathogenic *E. coli*, which enhances the expression of Stx2a. In this way, non-pathogenic *E. coli* decreases the expression of TJ proteins, increasing the production of the STx2a toxin by *E. coli* O157:H7 strain (Xiaoli et al., 2018). This indicates that, under certain conditions, even non-pathogenic microbiota can have a negative impact on intestinal wall permeability. Contrarily, the use of probiotics (living microorganisms that are beneficial to the host organism when administered in adequate amounts) may contribute to the integrity of the intestinal barrier (Ulluwishewa et al., 2011; Gou et al., 2022). In particular, *Lactobacillus* and *Bifidobacterium* species are the most commonly used probiotics. For example, *Lactobacillus reuteri* increases the expression of TJ proteins and thus supports the integrity of the intestinal wall (Gou et al., 2022). Oral administration of *L. reuteri I5007* significantly increased the levels of claudin-1, occludin, and ZO-1 in newborn piglets. An *in vitro* study showed that pretreatment of intestinal porcine epithelial cell line J2 with this bacterial strain suppressed a LPS-induced decrease in TJ protein expression (Yang et al., 2015). Administration of *L. plantarum* into the duodenum of healthy people increased the level of ZO-1 and occludin. However, *L. plantarum* did not significantly affect expression of occludin *in vitro* human epithelial model but induced translocation of ZO-1 into the TJ region which forms a paracellular seal between epithelial cells (Karczewski et al., 2010; Caminero et al., 2023). *Bifidobacterium infantis* and *L. acidophilus* prevented dysregulation of occludin and claudin-1 levels in colon carcinoma cell line (Caco-2) stimulated by IL-1β treatment. These strains normalized their expression and contributed to the integrity of the intestinal barrier (Guo et al., 2017). For convenience, we have summarized some available information regarding the influence of different probiotic bacterial strains on TJ proteins in Table 1. In general, probiotic bacteria can both increase and decrease TJ proteins. However, in most cases, this does not cause excessive intestinal permeability, but on the contrary, normalizes it and contributes to its integrity.

**TABLE 1:** Influence of probiotic bacteria on tight junction proteins.

Bacterial strain	Experimental subject	Tight junction protein increased (↑) or decreased (↓)	References
*Lactobacillus reuteri* I5007	Newborn piglets	Occludin, claudin-1, ZO-1 protein level ↑	Yang et al. (2015)
*L. reuteri*	Sprague–Dawley rats with acute liver failure	Occludin and ZO-1 expression ↑	Zhou et al. (2022)
*L. reuteri* DSM 17938 and 1563F	Enterotoxigenic *E. coli* -infected IPEC-J2 cells	ZO-1 expression ↑	Karimi eta. (2018)
*L. reuteri* ZJ617	LPS-injected C57black/6 mice	Occludin and claudin-3 expression ↑	Cui et al. (2017)
*L. plantarum* WCFS1	Duodenum of healthy people	Occludin and ZO-1 fluorescent intensity ↑	Karczewski et al. (2010)
*L. plantarum* WCFS1	Small intestine of healthy people	Claudin-5 expression ↓	Mujagic et al. (2017)
*L. plantarum* CIP48	Small intestine of healthy people	Claudin-19 expression ↓	Mujagic et al. (2017)
*L. plantarum* MB452	Caco-2 cell line	Occludin, ZO-1, ZO-2 fluorescent intensity ↑	Anderson et al. (2010)
*L. acidophilus*	IL-1β-induced Caco-2 cells	Occludin ↑ and claudin-1 expression ↓	Guo et al. (2017)
*L. rhamnosus* GG	LPS-injected C57black/6 mice	Occludin and claudin-3 expression ↑	Cui et al. (2017)
*Lactiplantibacillus plantarum* ST-III	Caco-2 cell line	ZO-1 expression ↑	Zhang et al. (2024)
*Lacticaseibacillus rhamnosus* KF7	Caco-2 cell line	Occludin and ZO-1 expression ↓	Zhang et al. (2024)
*Bifidobacterium infantis*	IL-1β-induced Caco-2 cells	Occludin ↑ and claudin-1 expression ↓	Guo et al. (2017)
*B. infantis*	IFN-γ or TNF-α-induced T84 cells	Occludin ↑, claudin-2 ↓, ZO-1 expression ↑	Ewaschuk et al. (2008)
*B. bifidum*	TNF-α-induced Caco-2 cells	Occludin expression ↑	Hsieh et al. (2015)

Abbreviations: ZO, zonula occludens; IPEC-J2, intestinal porcine enterocyte cell line derived from the jejunum of a neonatal piglet; LPS, lipopolysaccharides; Caco-2, cancer coli (human colorectal adenocarcinoma cells); IL-1β, interleukin 1β; T84, transplantable human carcinoma cell line; IFN-γ, interferon γ; TNF-α, tumor necrosis factor α.

Antibiotics used to treat bacterial infections may adversely affect the gut microbiota. They cause an imbalance between specific groups of bacteria and trigger the development of dysbacteriosis (Tulstrup et al., 2015). Dysbacteriosis, in turn, contributes to intestinal permeability. An increase in the population of pathogenic bacteria at dysbacteriosis which probably produce higher levels of LPS, can damage epithelial cells of the intestinal barrier and contribute to increased intestinal permeability. For example, it was shown that changes in the microbial composition correlated with an increase in intestinal permeability in alcohol-dependent subjects (Leclercq et al., 2014).

In addition, the gut microbiota is a significant source of digestive proteases used to break down host proteins for their own needs. However, excessive activity of microbial proteases can disrupt the epithelial components of the intestinal barrier due to cleavage of TJ proteins. In turn, changes in TJ proteins lead to an increase in the paracellular permeability of the epithelial barrier (Caminero et al., 2023).

### 3.2 The role of gut microbiota in biosynthesis and degradation of mucous layer components

The mucous layer separates gut microorganisms from the epithelium. Mucus consists of 95% water and the rest are mucin proteins. The latter assemble into long polymers that form a gel-like structure that can hold numerous bacteria. Mucins are highly glycosylated which allows them to maintain a high-water content in the mucus (Hansson, 2019).

The gut microbiota can modulate the properties of mucus. In particular, bacteria can affect the glycan profile of mucus via the activation of glycosyltransferases. For example, *Bacteroides* spp. induces expression of α1,2-fucosyltransferase that promotes fucosylation (Comstock and Kasper, 2006). In general, these enzymes create a selective habitat for the gut microbial community because only certain microorganisms can bind to the mucin glycans in mucus. These mucin glycans are the place of attachment for bacteria and contribute to their colonization (Paone and Cani, 2020). In this way, gut microbiota can change the composition and properties of mucus that undoubtedly affects the integrity and permeability of the intestinal barrier.

In addition, mucin glycans can be used by bacteria as an energy source. In the distal part of the intestine where there are not enough nutrients obtained from food, microorganisms actively utilize the mucus layer (Qu et al., 2021). Bacteria such as *Akkermansia muciniphila*, *Bacteroides thetaiotaomicron*, *Bifidobacterium bifidum*, and others are called mucolytic ones because they may degrade mucin. Such degradation is carried out by glycosidases (Sicard et al., 2017). For example, *Bacteroides* can secrete fucosidases that cleave the terminal fragments of fucose from glycans (Comstock and Kasper, 2006). Analysis of 374 gut microbiota genomes showed that 86% of the analyzed genomes contain genes necessary for the cleavage of mucin glycans and 89%—genes necessary for the catabolism of derived monosaccharides (Ravcheev and Thiele, 2017). This indicates that most microorganisms of the gut microbiota may degrade and metabolize mucin glycans. In addition, mucus that has undergone gut bacteria-induced degradation becomes less viscous and more permeable to toxins and microbes, which can facilitate the entry of luminal bacteria and cause increased inflammatory responses (Wang et al., 2021). However, alterations in the mucus layer can also affect the composition and biodistribution of the gut microbiota. In particular, the glycan repertoire of mucins determines which bacteria will grow. Certain bacteria can bind or degrade certain mucin glycans as a source of nutrients. Accordingly, bacterial degradation of mucin glycans can suppress the growth of other bacteria that have lost sites responsible for their binding to mucus. In turn, losing some bacteria can lead to excessive growth of others contributing to dysbacteriosis development (Schroeder, 2019).

### 3.3 Antioxidant effects of intestinal microorganisms

Intestinal barrier dysfunction is often accompanied by the development of oxidative stress. The latter is an imbalance between the generation and elimination of ROS in favor of the former with various consequences for cell physiology (Lushchak, 2014b). ROS are highly reactive substances concentration of which depends on the physiological state of the organism. Normally, much less than 10% of consumed oxygen is converted to ROS during functioning of the mitochondrial electron transport chain (Lushchak, 2014b). Inside the cell, there is a so-called basal steady-state (stationary) level of ROS. Under these conditions, visible harmful effects are not registered and this state is called basal intensity oxidative stress. When the level of ROS increases, low intensity, intermediate, and high intensity oxidative stress can occur (Lushchak, 2014a). For example, Banan et al. (2000) found that hydrogen peroxide in a dose-dependent manner disrupts the barrier function of monolayers of Caco-2 cells. In addition, there is a negative linear correlation between increased ROS generation and decreased paracellular barrier function (Elamin et al., 2013). Protection against ROS is provided by the antioxidant defense system, that can decrease the level of ROS and return it to the original range.

Probiotics are known for their many beneficial effects including antioxidant properties. Bacteria of the genera Lactobacillus and Bifidobacterium can neutralize ROS and increase the level of antioxidants (Wang et al., 2017). For example, *L. plantarum* 124 exhibits a strong antioxidant effect that is probably related to the secretion of a powerful antioxidant L-ascorbic acid (Wu et al., 2022). In addition, *L. plantarum* possesses an antioxidant enzyme called pseudocatalase that eliminates hydrogen peroxide (Feng and Wang, 2020). Furthermore, SCFAs which are produced by many bacteria can activate the Keap1-Nrf2 system in the host cells influencing the expression of antioxidant enzymes (Kunst et al., 2023). Thus, some representatives of the gut microbiota can reduce the level of ROS and prevent the development of oxidative stress. In turn, this has a positive effect on the integrity of the intestine and limits activation of the immune system.

Virtually all organisms possess antioxidant defenses and this has to be studied systematically. Evaluation of the intensity of free radical processes in the intestine needs a special approach. The presence of a huge oxygen gradient across the intestinal wall is a very important problem. It has been established that the apical mucosa closest to the lumen maintains *in vivo* oxygen concentrations of 0.1%–1%, whereas in the vascularized submucosa, oxygen concentration is ∼6%, or even more. The colonic muscle is the most oxygenated region with 7%–10% oxygen levels (Schwerdtfeger et al., 2019). Such an oxygen gradient requires cells, both microbial and host ones, to have a high capability for adapting to oxygen at different concentrations. Antioxidant defense plays a crucial role in surviving changes in oxygen levels. Hence, there is always a question of the efficiency of antioxidant defenses as measured under experimental conditions (*ex vivo*) as compared to conditions close to the natural environment of the GIT. In most cases, this issue has been totally ignored and experiments are run at an oxygen concentration of 21% that is the oxygen concentration in air. However, most intestinal microorganisms including *Lactobacillus* and *Bifidobacterium* are microaerophiles or even anaerobes. Thus, experimentally received data on the antioxidant potential of intestinal bacteria has to be treated accurately to reflect the real antioxidant properties of the gut microbiota (Lushchak, 2001).

## 4 Molecular mechanisms of the activation of pro-inflammatory response caused by bacterial invasion

Dysbacteriosis of the gut microbiota can lead to disruption of intestinal barrier function and immune homeostasis. Increased intestinal permeability facilitates the translocation of microbes, their components, and microbial products into the blood stream and their recognition by the host immune cells (Longo et al., 2020). The gut microbiota is the main reservoir of pro-inflammatory endotoxins inside the body. In particular, LPS, the main component of the outer membrane of Gram-negative bacteria, can cause so-called endotoxemia. The latter develops when the level of LPS in the blood increases and this leads to the activation of a pro-inflammatory immune response triggering systemic low-grade inflammation (André et al., 2019). A diet-induced increase in LPS concentration in the blood is called metabolic endotoxemia. The level of LPS in the blood serum of mice that consumed high-fat diet (HFD) for 4 weeks is similar to its level in metabolic endotoxemia (Mohammad and Thiemermann, 2021). This clearly shows how nutrition can affect intestinal permeability and immune response.

The dynamic interaction between the gut microbiota and the intestinal immune system plays a key role in maintaining intestinal homeostasis. Host cells contain pattern recognition receptors (PRRs) which recognize bacterial pathogen-associated molecular patterns (PAMPs). The latter are highly conserved bacterial motifs, possessed in LPS, oligodeoxynucleotides, peptidoglycans, and others that can trigger host immune response (Asiamah et al., 2019). PRRs are divided into two groups according to their location in the cell: intracellular receptors and transmembrane receptors. The nucleotide-binding oligomerization domain-(NOD-)-like receptors (NLRs) and retinoic acid-inducible gene-(RIG-)-like receptors (RLRs) belong to the first group (intracellular), whereas transmembrane receptors include Toll-like receptors (TLRs) and C-type lectin receptors (CLRs) (Jang et al., 2015). However, there are some exceptions. For example, TLR1, TLR2, TLR4, TLR5, TLR6, TLR10 are indeed located on the plasma membrane, whereas TLR3, TLR7, TLR8, TLR9, and TLR11 families are intracellular TLRs expressed in the membranes of endosomes and lysosomes (Lester and Li, 2014).

TLRs are highly expressed on the surface of epithelial cells (Semin et al., 2021). Their expression is tightly regulated to ensure proper recognition of bacterial PAMPs. There are two main ways to avoid excessive interaction of the gut microbiota with TLRs to prevent activation of the intestinal and in some cases systemic immune response: i) decreasing the expression of TLRs, and ii) moving TLRs from the plasma membrane into lysosomes (Semin et al., 2021). In addition, TLRs are expressed differently on the apical and basolateral membranes of epithelial cells. For example, TLR4 in humans are mainly expressed on the basolateral membrane of epithelial cells which does not come into contact with microbes. Less often, they are found on the apical membrane being constantly exposed to commensal microorganisms and microbial products that are ligands for specific TLRs (Yu and Gao, 2015). Thus, the different expression of receptors on the apical and basolateral membranes can be considered as a kind of molecular mechanism that allows to avoid of excessive stimulation by luminal microbes.

Some TLRs are involved in the regulation of intestinal epithelial renewal. In particular, Hörmann et al. (2014) suggested that increased proliferation of the small intestinal epithelial cell line MODE-K could be due to increased TLR2 signaling resulting in activation of the ERK1/2 and AKT pathways. Interestingly, TLR2-deficient mice demonstrate reduced epithelial cell proliferation (Hörmann et al., 2014). However, it is noteworthy that TLR2 was found to be involved in the weakening of the intestinal barrier mediated by commensal microbiota. In particular, TLR2 decreased the level of the epithelial transmembrane glycoprotein NRP1 (epithelial neuropilin-1) which is a positive regulator of Hedgehog (Hh) signaling. This results in the downregulation of Hh, which plays a pivotal role in gut development. Thus, the commensal microbiota can weaken the intestinal barrier through postnatal control of Hh signaling associated with TLR2-induced decreasing of NRP1 (Pontarollo et al., 2023).

In the present review, we will focus mainly on the signaling cascade of TLR4 because it is believed to be the main sensor of bacterial LPS (Ignacio et al., 2016). In addition, some studies link TLR4 with changes in the gut barrier function. In particular, increased intestinal permeability is associated with increased TLR4 expression. The latter upregulates expression of protein kinase C, contributing to the dephosphorylation of occludin, one of the key TJ proteins. As a result, the distribution of occludin within the intercellular junction complex decreases, the interstitial space expands, and endotoxins, including bacterial LPS, can enter the portal circulation (Li et al., 2013). Overall, enhanced TLR4 signaling is characterized by increased bacterial translocation and disruption of gut barrier function, while TLR4 deficiency alleviates intestinal barrier dysfunction (Dheer et al., 2016; Zhan et al., 2022).

### 4.1 Early/late activation of inflammation by TIRAP/MyD88 and TRAM/TRIF signaling cascades

**FIGURE 2:**
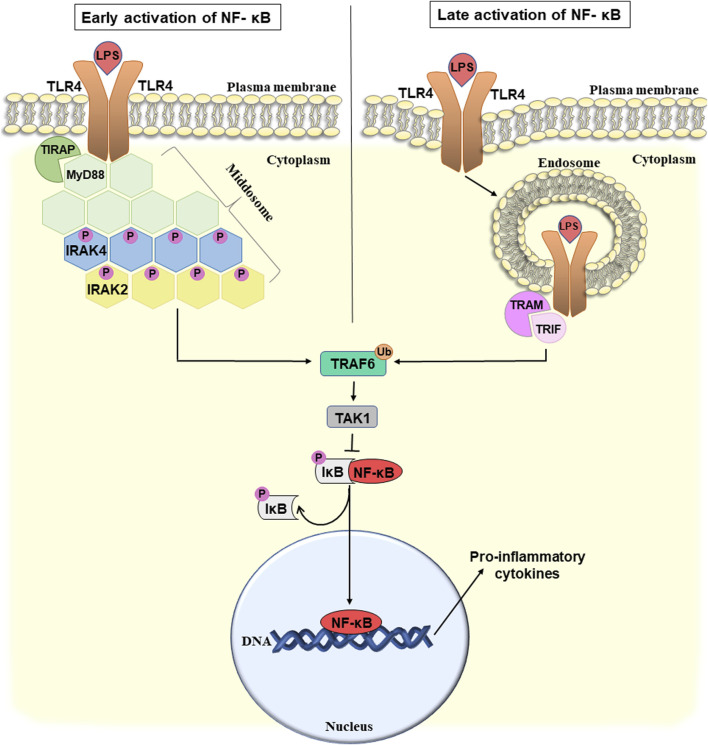
Potential mechanism of early and late activation of NF-κB-mediated pro-inflammatory response and bacterial translocation due to the disruption of the intestinal wall integrity. For details see the text. Abbreviations: NF-κB, nuclear factor κB; LPS, lipopolysaccharide; TLR4, toll-like receptor 4; TIRAP, toll/interleukin-1 receptor domain-containing adapter protein; MyD88, myeloid differentiation factor 88; IRAK 2/4, interleukin-1 receptor-associated kinase 2/4; TRAF6, TNF receptor-associated factor 6; TAK1, TGFβ-activating kinase 1; IκB, inhibitor of NF-κB; TRAM, TRIF-related adapter molecule; TRIF, TIR-domain-containing adapter-inducing interferon-β; P, phosphate; Ub, ubiquitin.

The interaction between PAMPs and TLRs takes place in several steps and requires the participation of many proteins. Initially, upon interaction with LPS, the receptor dimerizes. The formed aggregates are recognized by two pairs of adapter proteins TIRAP/MyD88 and TRAM/TRIF, that interact with cytoplasmic domains of the TLR4. The TIRAP/MyD88-induced signaling cascade begins from the plasma membrane, and TRAM/TRIF from the endosomes (Kagan et al., 2008). These are schematically depicted in Figure 2.

TIRAP (toll/interleukin-1 receptor domain-containing adapter protein) recruits MyD88 (myeloid differentiation factor 88) directly to the TLR4 cytoplasmic domain. Thus, MyD88 and TLR4 interact not directly but with the participation of TIRAP triggering the formation of a signaling complex. Next, MyD88 binds interleukin-1 receptor-associated kinase 4 (IRAK4), and promotes its autophosphorylation (Kuzmich et al., 2017). TLR4, MyD88, and IRAK4 form a complex called the middosome, that acts as a platform to recruit other members of the IRAK family, such as IRAK1 and IRAK2. IRAK4 is considered to be a catalytic protein kinase, that is first autophosphorylated and then sequentially activates IRAK1 and IRAK2 (Vollmer et al., 2017). The stoichiometric ratio of middosome components is six or eight molecules of MyD88, four molecules of IRAK4, and four molecules of IRAK1/2. It is believed that IRAK2 is the main protein recruited to the middosome and is responsible for late-phase TLR signaling. IRAK1 is recruited to the middosome only at an early stage of the signaling pathway and when IRAK4 kinase activity is insufficient. Assembly of the middosome induces association and activation of the E3 ubiquitin ligase TNF receptor-associated factor 6 (TRAF6) which can trigger several different pathways (Pereira and Gazzinelli, 2023). Here we will discuss one of them that is associated with activation of nuclear factor (NF)-κB-mediated pro-inflammatory response. Other TRAF6-activated pathways are associated, for example, with the induction of interferon production.

TRAF6 activation is associated with its autoubiquitination and subsequent recruitment of TGFβ-activating kinase 1 (TAK1) (Pereira and Gazzinelli, 2023). TAK1 phosphorylates the IκB (inhibitor of NF-κB) kinase complex that, in turn, leads to activation of the nuclear factor (NF)-κB pathway (Ouyang et al., 2014). NF-κB is a transcription factor, that in response to numerous stimuli, including bacterial LPS, triggers the development of inflammation. Normally, it is located in the cytoplasm in an inactive complex with its inhibitor, IκB. As a result of the phosphorylation of IκB, NF-κB is released from the inhibitory complex, translocates into the nucleus and activates the transcription of various genes involved in the immune response. NF-κB is a key regulator of pro-inflammatory cytokines including IL-6, IL-8, and others which contributes to the development of chronic inflammation (Israël, 2010).

Therefore, above we considered how TLR4 potentially can trigger LPS-induced inflammation caused by the disruption of intestinal integrity through the TIRAP/MyD88 signaling cascade, that leads to the early activation of NF-κB (Figure 2, left part). The TRAM/TRIF signaling cascade, which is also triggered by TLR4, provides late activation of NF-κB (Figure 2, right) (Nilsen et al., 2015).

TRAM (TRIF-related adapter molecule) acts as a bridge adapter between TRIF (TIR-domain-containing adapter-inducing interferon-β) and TLR4. TRIF mediates the activation of interferon 3 expression and plays an important role in the development of LPS-induced endotoxin shock (Fitzgerald et al., 2003). However, similar to TIRAP/MyD88, the TRAM/TRIF pathway can also lead to NF-κB activation. In particular, TRIF interacts with TRAF6, that then activates TAK1 and triggers the activation of NF-κB (Kawai and Akira, 2007).

Thus, bacterial LPS can induce a chronic inflammatory process triggering many diseases such as pathogenesis of Alzheimer’s disease, obesity, atherosclerosis, and others (Page et al., 2022). In addition, the gut microbiota is closely related to autoimmune diseases that arise as a result of the host’s immune system attacking host tissues.

The intestinal immune system normally tolerates commensal microorganisms (De Luca and Shoenfeld, 2019). However, dysbacteriosis can induce the development of such autoimmune diseases as multiple sclerosis or type I diabetes (Mousa et al., 2022). On the other hand, Gram-positive Lactobacilli and Bifidobacteria are considered to be anti-inflammatory bacteria (Sanders et al., 2021). For example, some Bifidobacteria strains do not activate NF-κB in the intestinal epithelial cells. Moreover, some of them inhibit LPS-induced TLR4-mediated NF-κB activation in a dose- or strain-dependent manner. For example, *L. plantarum* 1201 decreased intestinal inflammation in mice by downregulating proteins associated with the TLR4/NF-κB pathway (Ren et al., 2022). In this regard, they may be considered as candidates for probiotic intervention in chronic intestinal inflammation (Riedel et al., 2006). In this way, gut microorganisms can have both an anti-inflammatory effect and, conversely, cause the development of local and systemic inflammation. Because experimental data are received from different conditions, the data are not easily systematized. For example, it is not clear if Lactobacilli and Bifidobacteria act as antioxidants directly or if their effects are mediated by some proteins or small molecules.

## 5 Nutrition as regulator of intestinal barrier permeability

The integrity of the intestinal barrier plays a crucial role in the health of the whole organism. Changes in intestinal permeability are connected with many pathologic conditions. Disturbance of the gut microbiota is involved in the development of Parkinson’s disease (Dwyer et al., 2021), obesity, diabetes, inflammatory bowel diseases, chronic liver diseases, neuropsychiatric disorders, and others. Specially designed nutrition may be an effective way to protect and restore the integrity of the intestinal barrier and recreate a healthy microbiota composition (Inczefi et al., 2022).

In general, dairy products are among the most consumed foods in humans. Numerous studies show a connection between fermented dairy products and improved health due to stimulation of the growth of beneficial bacteria because they contain diverse microbial communities, where lactic acid bacteria usually dominate (Jatmiko et al., 2018). For example, oral administration of heat-treated *Lactobacillus plantarum* OLL2712 prevented the downregulation of ZO-1 and occludin in HFD-fed mice, promoting intestinal barrier integrity (Wang et al., 2023). Enteral administration of *Lactobacillus plantarum* 299 for 16 days to rats with experimental biliary obstruction decreased intestinal permeability (White et al., 2006). Furthermore, pretreatment of intestinal porcine epithelial cell J2 cultures with *L. plantarum* ZLP001 counteracted to the increase in intestinal permeability induced by enterotoxigenic *Escherichia coli* via prevention of a decrease in the level of TJ proteins (claudin-1, occludin and ZO-1) (Wang et al., 2018). Thus, the consumption of fermented dairy products that contain a significant amount of beneficial lactic bacteria can potentially prevent and restore disruption of intestinal integrity and related diseases.

Some dairy products, such as kefir, have long been studied as regulators of intestinal integrity. For example, consumption of kefir for 21 days by healthy people with two washout periods in-between decreased the serum level of zonulin (Novak et al., 2020) Kefir diet also normalized zonulin level in overweight people (Pražnikar et al., 2020). Zonulin is a protein that increases the permeability of the intestinal barrier and is often involved in the development of autoimmune diseases, including type I diabetes. Zonulin causes TJ disassembly and thus violates the intestinal barrier (Fasano, 2011). Therefore, zonulin is considered a serum marker of the integrity of the intestinal wall.

Polyphenolic compounds (secondary plant metabolites with a long list of beneficial properties for humans) are other food components that improve intestinal integrity. Flavonoids are among most abundant representatives of this group. They are found mostly in fruits, vegetables, grains, tea, and wine (Kasprzak-Drozd et al., 2021). For example, the flavonoid quercetin increased intestinal integrity, as studied in Caco-2 cells (Suzuki and Hara, 2009). This effect was associated with the assembly of ZO-2, occludin and claudin-1, as well as increased expression of claudin-4 and transepithelial electrical resistance. The electrical resistance of epithelial cells is a reliable indicator of the integrity and permeability of the cell monolayer and TJ (Srinivasan et al., 2015). The consumption of quercetin in food increased the mRNA levels of occludin and ZO-1 in pigs that was accompanied by a decrease in serum endotoxin (Zou et al., 2016), a marker of metabolic endotoxemia frequently associated with increased intestinal permeability. The flavonoid kaempferol may have similar effects. In a study on Caco-2 cells during the first 6 hours after kaempferol administration, transepithelial electrical resistance increased significantly and this correlated with the assembly of occludin and claudin-3 (Suzuki et al., 2011).

A meta-analysis performed to study the effects of oral administration of phenolic compounds on the integrity of the intestinal barrier in animals confirmed their beneficial effects. In particular, the improvement of intestinal wall integrity occurs due to the three main mechanisms: i) increased expression of TJ proteins, ii) decreased levels of pro-inflammatory molecules, and iii) increased intracellular antioxidant potential (Sandoval-Ramírez et al., 2021).

In addition to phenolic substances, plant food also contains other useful phytocompounds positively affecting gut barrier function. For example, sulforaphane, an isothiocyanate common in Brassicaceae family, protects the mucous membrane of GIT from oxidative damage through the activation of the Nrf2-Keap1 system, upregulating the transcription of antioxidant enzymes (Yanaka, 2017). In addition, sulforaphane normalizes gut microbiota dysbiosis and increases the expression of TJ proteins, contributing to intestinal homeostasis (He et al., 2018). For convenience, we have structured some information regarding the influence of the above-mentioned phytocompounds on the gut barrier function in Table 2.

**TABLE 2:** Influence of phytocompounds (quercetin, kaempferol, and sulforaphane) on gut barrier function.

Phytocompounds	Experimental subject	Effect on gut barrier function	References
Quercetin	Caco-2 cell line	TER, claudin-4 expression ↑	Amasheh et al. (2008)
Quercetin	Caco-2 cell line	TER, claudin-4 expression, assembly of ZO-2, occludin, and claudin-1 ↑	Suzuki and Hara (2009)
Quercetin	Villus epithelium	TER, claudin-4 protein level ↑; claudin-2 protein level ↓	Cornelius et al. (2024)
Kaempferol	Caco-2 cell line	TER, assembly of ZO-1, ZO-2, occludin, claudin-1, claudin-3, and claudin-4 ↑	Suzuki et al. (2011)
Kaempferol	LPS-induced epithelial-endothelial cells co-culture	TER, occludin, claudin-2 and ZO-1 expression ↑; IL-8 ↓	Bian et al. (2020)
Kaempferol	C57black/6 mice fed a high-fat diet	Gut microbiota dysbiosis, TNF-α protein level, intestinal permeability ↓	Bian et al. (2022)
Sulforaphane	LPS-induced Caco-2 cell line	TER, SOD, GPx, CAT activities ↑; IL-1β, IL-6, IL-8 і TNF-α levels ↓	Zhang and Wu (2021)
Sulforaphane	C57black/6 mice with bladder cancer	Gut microbiota dysbiosis, pathological signs in colon ↓; claudin-1 expression ↑	He et al. (2018)
Sulforaphane	C57black/6 mice fed a high-fat and high-fructose diet	ZO-1, claudin-4 expression ↑; TLR4, Myd88, NF-κB expression ↓	Xu et al. (2023)
Sulforaphane	C57black/6 mice with ulcerative colitis	Gut microbiota dysbiosis, damage scores of colon ↓	Zhang et al. (2020)

↑, increased; ↓, decreased. Abbreviations: Caco-2, cancer coli (human colorectal adenocarcinoma cells); TER, transepithelial resistance; ZO, zonula occludens; LPS, lipopolysaccharides; IL, interleukin; TNF-α, tumor necrosis factor α; SOD, superoxide dismutase; GPx, glutathione peroxidase; CAT, catalase; TLR4, Toll-like receptor 4; Myd88, myeloid differentiation factor 88; NF-κB, nuclear factor κB.

In general, nutrition is considered to be among the most important factors influencing health. Consumption of certain foods can prevent the development of numerous pathologies. However, over-emphasis on any one nutrient or monotonous food is not recommended (Katz and Meller, 2014), since the lack of variety in dietary products can cause malnutrition and imbalance in consumed nutrients. For example, the monotonous cereal diet characteristic of the poorest households in Sri Lanka at least demonstrates a lack of necessary micronutrients (Weerasekara et al., 2020). Whereas the consumption of a variety of food products, that is, dietary diversity, usually is useful for health. However, under certain conditions at some pathologies, a monotonous diet can be beneficial. For example, a complete monotonous diet (containing all required micro- and macronutrients) alleviates the severity of colonic inflammation in mice with acute colitis (Nagy-Szakal et al., 2013).

The choice of diet should take into account the balance of all required nutrients. For example, HFD has a negative effect on gut integrity. Consumption of HFD by rats for a month decreased the expression of TJ proteins and increased damage to the colon resulting in an increase in serum LPS (Chompre et al., 2022). HFD negatively affects the gut microbiota, enhancing the microbial ability to produce ROS. This is considered to be one of the key mechanisms for increasing the permeability of its barrier. It is worth to note, the HFD-induced ability to induce oxidative stress in the gut can be transferred by transplantation of fecal microbiota into germ-free mice (Zeng et al., 2023).

Thus, nutrition can affect the integrity of the intestine and this is often associated with various pathological conditions. The effect mainly occurs at the level of modulation of gut microbiota composition and regulation of TJ protein operation. In this regard, healthy nutrition can be considered as a promising way to attenuate various pathologies.

## 6 Exercise as a regulator of intestinal barrier integrity

Regular moderate physical exercises are one of the most common recommendations for the prevention of various pathologies, including disruption of the integrity of the intestinal barrier. This may be due to the influence of the gut microbiota. In particular, exercises have been found to increase gut bacterial diversity (Hintikka et al., 2023). However, effects of physical exercises depend on their intensity. For example, endurance athletes have a high incidence of gastrointestinal disorders and the “leaky” gut is one of the most common disorders (Ribeiro et al., 2021). It is characterized by dysfunction of the intestinal epithelial barrier and its excessive permeability. This results in penetration of harmful microorganisms, toxins or undigested food particles into the bloodstream and has a negative effect on health of the whole organism (Aleman et al., 2023).

The effect of exercise on intestinal permeability depends on its duration and intensity. For example, people who exercise frequently and intensely have the same mortality rates as people who lead a sedentary lifestyle (Van Houten et al., 2015). A 60 min bout of intensive treadmill running increased the permeability of the small intestine in runners, whereas low-intensity running had no such effect (Pals et al., 1997). Using the overtraining model with male C57BL/6 mice, it was established that exhaustive exercise exacerbated intestinal inflammation, disrupted integrity and enhanced intestine wall permeability (Hou et al., 2020). Sustained strenuous exercise in racing sled dogs increased the intestinal permeability and the frequency of gastric erosions or ulcerations (Davis et al., 2005). High-intensity interval running increased intestine wall permeability and intestinal-fatty acid binding protein (I-FABP) release in male runners (Pugh et al., 2017). I-FABP is a cytoplasmic protein expressed exclusively in the enterocytes of the small intestine and its increased concentration in the blood is used as a marker of damage to intestinal epithelial cells (Sikora et al., 2019).

Physical exercise of low/moderate intensity can often have positive effects and can be considered as a method of non-pharmacological intervention in inflammatory bowel disease (Ordille and Phadtare, 2023). For example, mice that swam for 30 min before inducing intestinal barrier dysfunction had less intestinal dysfunction compared to mice that had not swum before. This might happen due to a strengthening of antimicrobial function of the intestine as a result of the increase in expression of antimicrobial peptides (Luo et al., 2014). Obese mice that were trained on a motorized treadmill for 45 min per day 5 days a week for 12 weeks had higher expression levels of colonic ZO-1 and occludin. Moderate exercise effectively prevented the development of dysbacteriosis caused by the HFD, as well as intestinal pathology (Wang et al., 2022). Dysbacteriosis and impaired intestinal barrier integrity induced by HFD in wild type mice was prevented by exercise. Exercise on a motor-driven rodent treadmill for 5 days a week for a total of 15 weeks significantly reversed the pathological changes. Ablation of Sestrin 2 protein attenuated the protective effects of exercise, suggesting its involvement in regulation of intestinal permeability (Yu et al., 2022).

Thus, it can be concluded that high-intensity exercises often have a negative effect on the integrity of the intestine, whereas low- and moderate-intensity regular exercise can have a positive effects. It may be speculated that moderate damage to the intestinal wall is a hormetic factor that may be used to train organisms to cope with severe damaging challenges. This may be used to increase the adaptive potential of organisms to prevent damaging effects of any stresses of physical and chemical nature on the integrity of the intestinal wall.

### 6.1 Exercise-induced heat stress

It is known that physical exertion causes heat stress and associated dysfunction of gut integrity. A systematic review examining the relationship between an exercise-induced increase in core body temperature and intestinal permeability demonstrated that the magnitude of exercise-induced hyperthermia correlated with increased intestinal permeability (Pires et al., 2017). An increase in body temperature is a signal to activate the expression of heat shock proteins (HSP) which constitutively function as molecular chaperones maintaining the native structure of the proteins. Their expression is mainly triggered by heat shock signals. During exercise, the level of HSP70 and HSP90 increase (Krüger et al., 2019). Expression of HSP is regulated at the level of heat shock factors (HSF) such as HSF1 that is expressed in all mammalian tissues. Normally it resides in the cytoplasm as a monomer. In response to stressful conditions, it trimerizes, translocates into the nucleus, binds to the heat shock element of target genes and activates the transcription of HSPs, including HSP70/90 (Noble and Shen, 2012).

In this way, exercises cause a homeostatic imbalance, while regular training is adaptive and decreases the degree of this imbalance. Potentially, a higher adaptive steady-state level of HSPs due to regular training could explain their positive effect on gut integrity. At that time, during acute physical exertion, HSPs probably cannot cope with that level of homeostatic imbalance caused exercise-induced heat stress.

### 6.2 Exercise-induced hypoxia

**FIGURE 3:**
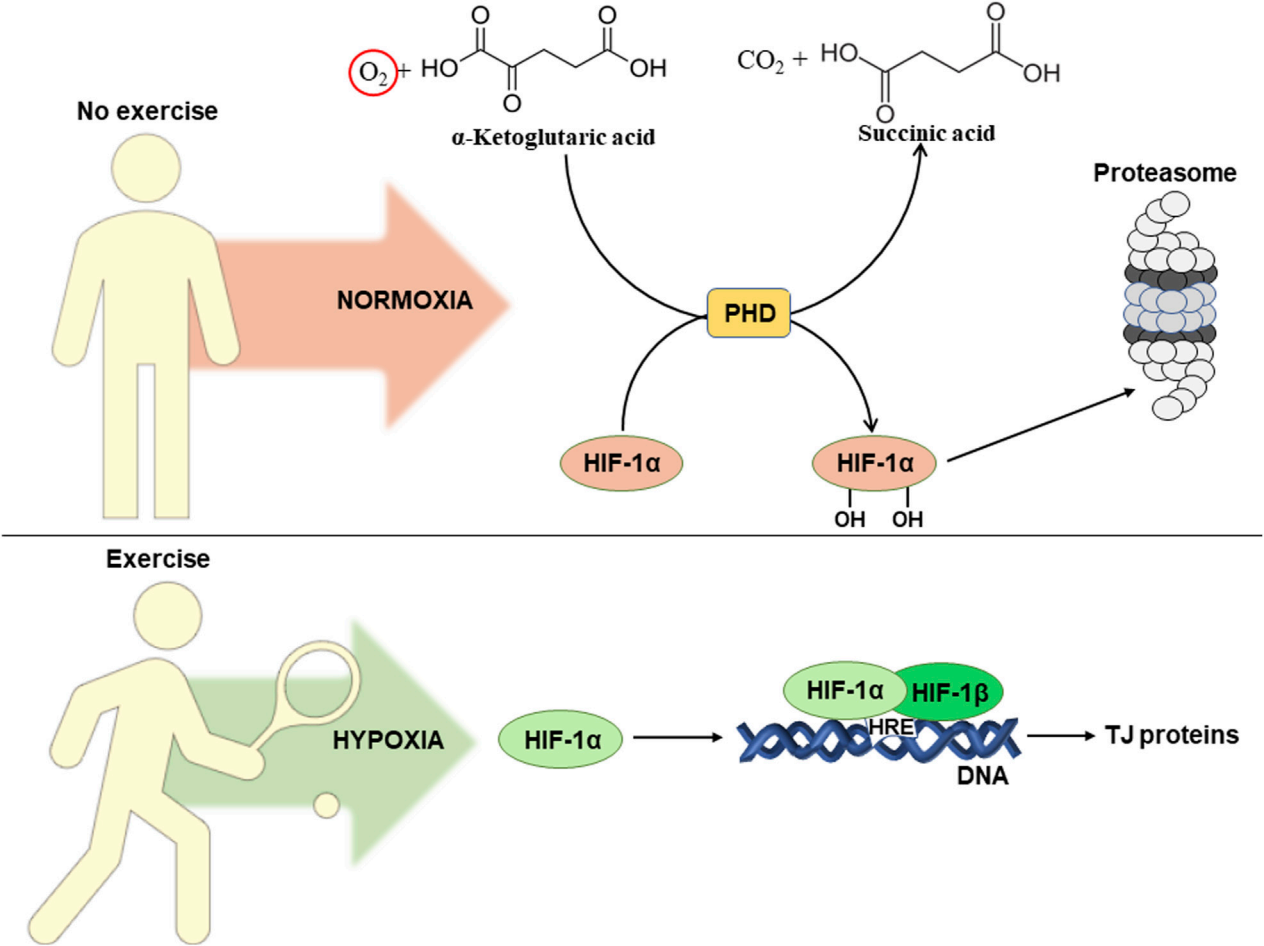
Influence of exercise-induced hypoxia on intestinal permeability. For details see the text. Abbreviations: HIF-1α/β, hypoxia-inducible factor 1α/β; HRE, hypoxia responsive element; DNA, deoxyribonucleic acid; TJ, tight junction; PHD, prolyl hydroxylase domain protein.

It is well known, that exercise causes a redistribution of blood flow between tissues. This leads to the development of hypoxia (decreased oxygen levels) in intestinal epithelial cells and activation of hypoxia-inducible factor alpha (HIF-1α) (Wu et al., 2020). Figure 3 schematically shows the influence of exercise-induced hypoxia on intestinal permeability. In normoxia (normal oxygen levels), prolyl hydroxylase hydroxylates HIF-1α at two proline residues (Pro 402 and Pro 564). This results in ubiquitination followed by subsequent proteasomal degradation of HIF-1α (Lee et al., 2004).

Due to lack of oxygen, hypoxia leads to inhibition of prolyl hydroxylase, which is also referred as prolyl hydroxylase domain (PHD) protein. Its activity decreases due to insufficient concentrations of oxygen, that is a co-substrate in the reaction of hydroxylation of HIF-1α (Epstein et al., 2001). This results in HIF-1α translocation into the nucleus, where in a complex with HIF-1β it activates transcription via binding to hypoxia responsive elements (HRE) in the promoters of corresponding genes (Kim et al., 2021). HIFs contribute to the adaptation of intestinal epithelial cells to hypoxic conditions. On the other hand, overactivation of this pathway can lead to intestinal damage and the development of inflammation. Thus, it is unclear whether regular exercises can really have a positive effect on intestinal permeability. Probably, the final result depends on the number of factors that will be considered below.

There are at least two HIF isoforms, HIF-1α and HIF-2α. HIF-1α is known to activate the transcription of genes that are responsible for strengthening of intestinal barrier function, protective immune response, and antimicrobial response. Meanwhile, regular activation of HIF-2α activates a pro-inflammatory response that causes intestinal damage (Shah, 2016). To our best knowledge, there are no data available on the involvement of HIF-2α in exercise-induced hypoxia. However, HIF-1α is considered as a potential regulator of intestinal permeability during regular exercise-induced hypoxia (Yu et al., 2022). In particular, it promotes intestinal integrity by activating the transcription of the TJ protein claudin-1. Its overexpression in HIF-1β-deficient intestinal epithelial cells leads to restoration of barrier function, confirming a crucial link between HIF and TJ function in the intestine (Saeedi et al., 2015). It has been established that regular aerobic exercise increases HIF-1α expression in HFD-induced intestinal dysfunction. This correlated with increased expression of TJ proteins, including ZO-1, occludin, and claudin-1 (Yu et al., 2022). Thus, HIF-1α could potentially be involved in improving gut integrity during hypoxia in epithelial cells as caused by regular exercise.

It is worth to note one important aspect of hypoxia-induced damage to the intestinal wall that seems has been overlooked. This is hypoxia-induced oxidative stress. Using a fish model, we previously demonstrated that hypoxia resulted in the development of oxidative stress (Lushchak and Bagnyukova, 2007). Later that phenomenon was confirmed with different models and was also implicated in human disorders (Lushchak et al., 2005; Pialoux and Mounier, 2012). We associated this with increased levels of electrons in mitochondrial electron-transport chain (ETC) under hypoxia as a result of limited access to molecular oxygen. In this case, electrons can escape the ETC and join O_2_ giving rise to ROS that leads to the development of oxidative stress. Generated ROS may cause damage to cells including epithelial ones and cell junctions leading to leakage of diverse intestinal components into the internal organismal milieu.

## 7 Conclusion and perspectives

The intestinal wall is a kind of checkpoint between the external and internal environments of organisms. The wall consists of three layers: mucous, epithelial, and lamina propria. The mucous layer is inhabited by microorganisms, many of which mutually beneficially coexistence within the human body. These microorganisms modulate many if not most living processes: from the development of the immune and nervous systems at early stages of life to the induction of chronic inflammation causing neurodegeneration at aging. Despite the fact that these microorganisms have coexisted with humans for many years, under certain conditions the enteral immune system of the lamina propria can perceive them as foreign and trigger a pro-inflammatory response.

Normally, the intestinal mucosa is semipermeable. It allows selective absorption of nutrients into the bloodstream but prevents the entrance of potentially harmful microorganisms and their waste products from contact with the enteral immune system. An imbalance of the intestinal microbiota, called dysbiosis, can cause a disturbance of intestinal integrity and increase intestinal permeability. Conversely, a healthy composition of the gut microbiota can contribute to the integrity of the intestinal barrier due to increased expression and induction of the assembly of TJ proteins, activation of mucus synthesis, and antioxidant action.

Disruption of intestinal barrier function may trigger development of local and even systemic inflammation. As a result, bacterial LPSs become available to immune cells of the host, recognized by PPRs on their surface, and activate a pro-inflammatory immune response. In particular, the role of TLR4 is well known and in response to LPSs can trigger early and late activation of the pro-inflammatory NF-κB transcription factor via TIRAP/MyD88 and TRAM/TRIF signaling cascades, respectively. In general, a vicious cycle of intestinal barrier disruption can be traced here, as excessive intestinal wall permeability provokes the development of chronic low-grade inflammation. The latter is characterized by increased production of pro-inflammatory cytokines and enhanced ROS generation, increasing intestinal barrier dysfunction.

Nutrition looks to be the simplest non-pharmacological effector of integrity and permeability of the intestinal wall. It can have both a negative effect, such as HFD inducing metabolic endotoxemia, or a positive effect, such as a diet rich in plant polyphenols or fermented dairy products, increasing the expression of TJ proteins and promoting the development of beneficial bacteria.

Exercise also can affect gut intestinal permeability. Its effects depend on duration and intensity of exercise. Acute extensive physical exertion often increases intestinal permeability which may be related to the induction of heat stress, that organisms cannot cope with at that time due to insufficient resources. On the other hand, regular low and moderate intensity exercises, that are adaptive in nature, mostly have a positive effect on the integrity of the intestine and decrease its permeability. Potentially, this may be associated with an increase in the steady-state level of HSPs and chronic activation of HIF-1α which activates the transcription of genes responsible for strengthening the intestinal barrier function.

**FIGURE 4:**
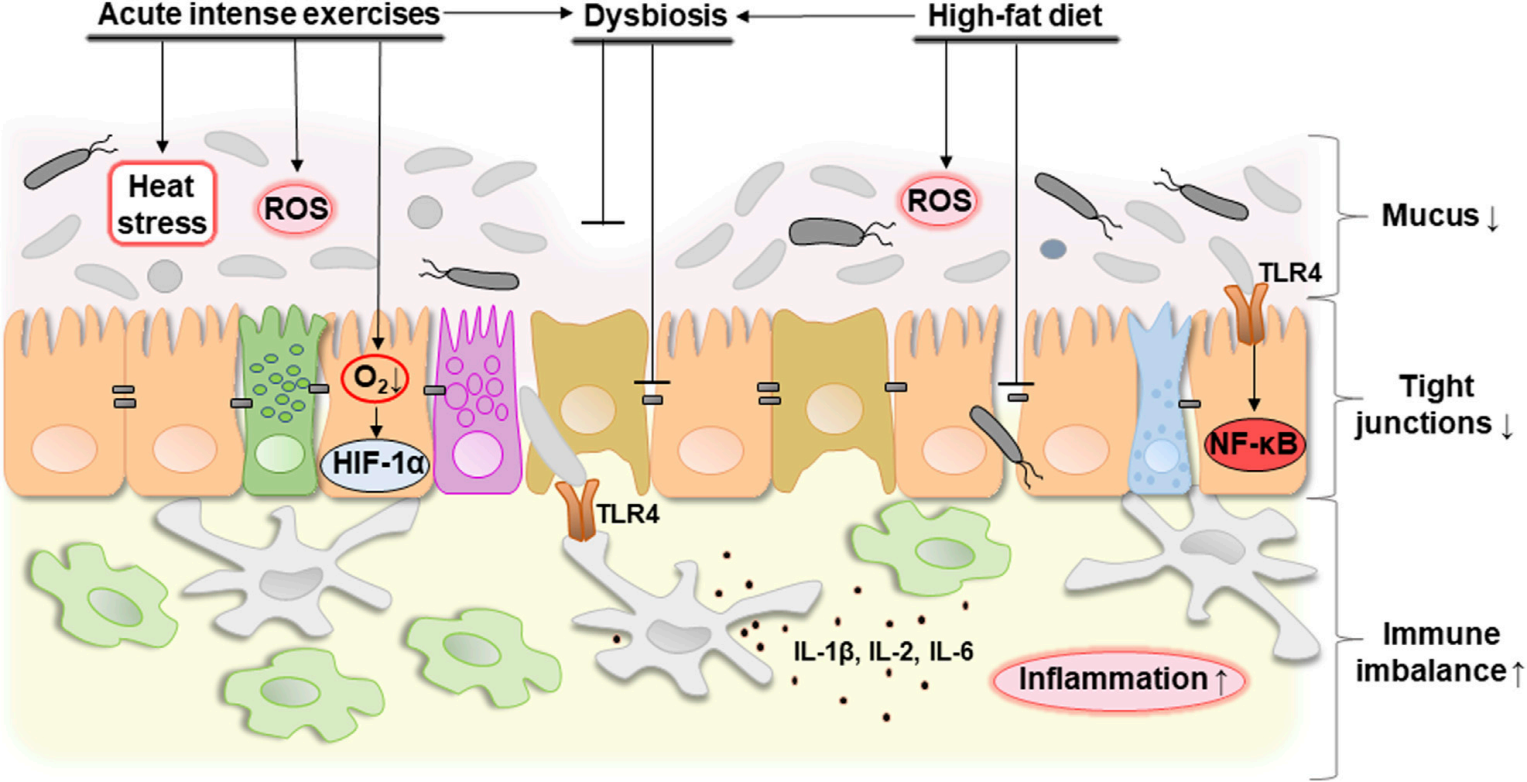
Potential consequences of acute intense exercise, unhealthy diet (e.g., high-fat diet) or dysbiosis on the intestinal barrier. Acute intense exercise contributes to the development of heat stress that the body cannot cope with, as well as a decrease in oxygen concentration, which causes hypoxia and the associated activation of HIF-1α. At the same time, hypoxia-related oxidative stress caused by an increase in the level of ROS can be observed. In general, these changes can cause the development of dysbacteriosis that can be associated with decreased expression of tight junction proteins and mucus degradation probably due to overgrowth of mucolytic bacteria. A high-fat diet also contributes to a decrease in tight junction proteins in combination with an increase in ROS production and the development of dysbacteriosis. In general, this contributes to a decrease in mucus thickness, an increase in the distance between neighboring epithelial cells and, as a result, an increase in the permeability of the intestinal barrier. This causes the development of an immune imbalance. Bacterial lipopolysaccharides are available as a result of increased permeability of the intestinal barrier and are recognized by TLR4 of the host’s epithelial and immune cells. This leads to the activation of NF-κB and production of pro-inflammatory cytokines such as IL-1β, IL-2, IL-6 triggering the development of inflammation. Abbreviations: ROS, reactive oxygen species; HIF-1α, hypoxia-inducible factor 1α; TLR4, toll-like receptor 4; NF-κB, nuclear factor κB; IL-1β/2/6, interleukin 1β/2/6. ↓, decreasing; ↑, increasing. Details of the structure of the intestinal barrier are shown in Figure 1.

In general, it can be concluded that proper nutrition which promotes a healthy biodiversity of the gut microbiota, combined with moderate exercise, contribute to the integrity of the intestine. Disbalanced nutrition and excessive physical activity can provoke the development of dysbacteriosis and increase intestinal permeability which can potentially lead to a pro-inflammatory response. Figure 4 schematically shows potential consequences of acute intense exercises, unhealthy diet (e.g., high-fat diet), and dysbiosis on the intestinal barrier.

Taking into account all of the above, we can outline the following future prospects:

1. Development of healthy diets to support intestinal homeostasis;
2. Use of fermented dairy products as natural pre-, pro- and postbiotics to promote a healthy gut;
3. Selection of exercises to promote intestinal integrity by frequency, intensity and duration;
4. Study of the role of intestinal HIF-2α during exercise;
5. Systemic investigation of hypoxia-induced oxidative stress as a regulator of intestinal wall permeability.

Most of these perspective avenues are directed to enhance the capability of organisms to cope with disturbing factors. That increases an adaptive capability via preadaptation/hormetic mechanisms. However, some of them may be used “to patch holes” in “leaky” intestinal wall, which is characterized by increased specific permeability of the intestinal epithelium.
